# Is the Risk of Diabetes Lower in Patients With Atrial Fibrillation Treated With Direct Oral Anticoagulant Compared to Warfarin?

**DOI:** 10.3389/fcvm.2022.874795

**Published:** 2022-05-19

**Authors:** Xuyang Liu, Shenghui Feng, Zhuohui Chen, Yue Zhou, Kang Yin, Zhengbiao Xue, Wengen Zhu

**Affiliations:** ^1^Department of Cardiology, Jinggangshan University, Ji’an, China; ^2^Queen Mary School, Medical Department, Nanchang University, Nanchang, China; ^3^Department of Cardiology, The First Affiliated Hospital of Sun Yat-sen University, Guangzhou, China; ^4^State Key Laboratory of Ophthalmology, Zhongshan Ophthalmic Center, Sun Yat-sen University, Guangzhou, China; ^5^Department of Critical Care Medicine, The First Affiliated Hospital of Gannan Medical University, Ganzhou, China

**Keywords:** atrial fibrillation, non-vitamin K antagonist oral anticoagulants, diabetes mellitus, warfarin, meta-analysis

## Abstract

**Background:**

The use of anticoagulants is an established strategy to prevent stroke, embolism, and cardiovascular mortality in patients with atrial fibrillation (AF), but its role in the prevention of incident diabetes is unclear. We aimed to investigate this question by using participant data from cohort studies.

**Methods:**

We conducted a meta-analysis of participants to investigate the impact of direct oral anticoagulants (DOACs) on the risk of new-onset diabetes in AF patients. The collection of related data was performed in the PubMed and EMBASE databases until December 2021, including studies associated with evaluating the correlation between DOACs and incident diabetes. The hazard ratios (HRs) and 95% confidence intervals (CIs) were adjusted by the random-effects model with an inverse variance method.

**Results:**

Two cohort studies with a total of 24,434 patients were included in this study (warfarin: *n* = 6,906; DOACs: *n* = 17,528). Compared with warfarin, the use of DOACs could reduce the incident diabetic risk in AF patients (HR = 0.75, 95%CI: 0.68–0.82). Investigations about the effects of three major classes of DOACs showed that the individual use of dabigatran (HR = 0.76, 95%CI: 0.64–0.90), rivaroxaban (HR = 0.74, 95%CI: 0.64–0.87), apixaban (HR = 0.74, 95%CI: 0.60–0.92) and the combined use of rivaroxaban and apixaban (HR = 0.74, 95%CI: 0.66–0.84) could reduce the risk of new-onset diabetes compared with warfarin. This risk reduction effect could be observed in both male and female groups (HR = 0.73, 95%CI: 0.64–0.84, *P* < 0.00001; HR = 0.82, 95%CI: 0.82–0.99, *P* = 0.04).

**Conclusions:**

Treatment with DOACs compared with warfarin reduced the risk of new-onset diabetes in both male and female patients with AF.

## Introduction

Atrial fibrillation (AF) is the most common cardiac arrhythmia in the clinic, characterized by high rates of thromboembolic complications and related mortality.

An integrated approach of AF care mainly contains rhythm and rate control therapy, anticoagulation therapy (OAC), and comprehensive upstream therapy (1). Among them, OAC is of vital importance in AF-related stroke prevention. As a traditional oral anticoagulant, vitamin K antagonists such as warfarin plays their role by antagonizing vitamin K epoxide reductase complex. Warfarin has a narrow therapeutic range, multiple drug and food interactions, and requires frequent blood monitoring of the international normalized ratio (INR) (2). Therefore, direct oral anticoagulants (DOACs, sometimes referred to as non-vitamin K antagonist anticoagulants) have been introduced in the clinic, reducing the risk of stroke or systemic embolism and bleeding compared with warfarin among patients with AF (3–5).

Patients with comorbid AF and DM have a higher risk of stroke, thromboembolism, and cardiovascular mortality (3, 6–9). Vitamin K has been suggested to regulate the activity of vitamin K-dependent proteins (VKDP) such as osteocalcin, which effectively improve β cell proliferation and insulin secretion to reduce the risk of new-onset DM (9–14). Due to the different effects of warfarin and DOACs on vitamin K, the use of DOACs was considered with the potential of reducing DM risk in AF patients. Two cohort studies have been conducted to compare the risk of DM induction in AF patients treated by warfarin and DOACs (9, 15). The study of Cheung et al. included the data of 13,688 DOACs new users from the Clinical Data Analysis and Reporting System (CDARS) managed by the Hong Kong Hospital Authority (HA). Their results showed that dabigatran was significantly related to incident diabetes risk reduction, and for Xa inhibitor anticoagulants, only the combination use of rivaroxaban and apixaban rather than individual drugs could decrease this risk. In this study, males were the only gender with a diabetic risk-reduction effect. While in a cohort analysis performed by Huang et al., a total of 10,746 AF patients from Taiwan’s National Health Insurance Research Database (NHIRD) were fitted into the study. All of the three different DOACs (dabigatran, rivaroxaban, apixaban) were reported with the incident diabetic reduction effect compared with warfarin. In this study, a similar trend of lowering new-onset DM risk was observed in both male and female groups treated with DOAC vs. warfarin. We used individual participants’ data from the two cohort studies to assess the impact of DOACs on new-onset diabetes risk in AF patients.

## Methods

The preferred reporting items for systematic review and meta-analysis (PRISMA) 2020 guidelines were used to conduct our present meta-analysis. Only published publications were included in our meta-analysis, so we did not need ethical permission. Readers can contact the corresponding authors for data, techniques, and materials to recreate the results or the program.

### Literature Retrieval

Two databases PubMed and Embase were used for systemic search in this study, retrieval keywords included (1) atrial fibrillation OR AF AND (2) incident diabetes OR new-onset diabetes AND (3) Direct oral anticoagulants OR DOAC OR DOAC OR oral anticoagulants OR dabigatran OR rivaroxaban OR apixaban AND (4) vitamin K antagonists OR warfarin OR VKA.

### Inclusion and Exclusion Criteria

The literature inclusion criteria of this study include (1) randomized controlled trials or observational cohort studies focusing on the risk of developing DM in AF patients treated by warfarin vs. DOACs (Apixaban, dabigatran, rivaroxaban or edoxaban), (2) The outcomes of studies include the appearance of new-onset diabetes, which meets the International Classification of Diseases, the use of anti-diabetic medication, or death occurred during the investigation period. (3) All of the patients included in the cohort study were treated with at least one type of anticoagulant after AF diagnosis. The clinical follow-up time was unlimited. Specific literature forms including reviews, case reports, case series, editorials, meeting abstracts, and insufficient clinical data were excluded.

### Study Selection and Data Extraction

Two independent researchers extracted data independently through screening the titles and abstracts to select potential studies for meta-analysis. Then full-text screening was carried out subsequently. Controversies were resolved by discussing with the third researcher. If multiple screened studies suitable for meta-analysis were from the same data source, the study that was more in line with predefined criteria was included. Studies with later publication years and longer follow-up times were preferentially included. The relevant information of each available study included the first author, publication year, study design, outcomes, types of DOACs, follow-up period, the sample size and the number of events in the warfarin or DOACs groups, hazard ratio (HR) and 95% confidence intervals (CI) were collected independently by the fourth author.

### Study Quality Assessment

Newcastle-Ottawa Scale (NOS) was used by authors to assess the quality of included studies. A total of nine points were divided into three domains, including the cohort selection (0–4 points), cohort comparability (0–2 points), and the outcomes evaluation (0–3 points) were assessed by the NOS tool. Studies with the NOS results < 6 points were considered as low quality.

### Statistical Analysis

We chose the Cochrane Q-test and I^2^ statistic to assess the consistency of the included studies. A *P* < 0.1 for the Q-test or I^2^ ≥ 50% result was considered as the existence of substantial heterogeneity. The Review Manager Version 5.3 (The Nordic Cochrane Center, The Cochrane Collaboration, 2014, Copenhagen, Denmark^1^) was used for all statistical analyses; *P* < 0.05 was considered statistically significant. First, we collected the sample size and number of events in the warfarin or DOACs groups, then the crude events rates of DM induction risk were carried out and expressed by HRs and 95% CIs. Second, the HR of DM induction was calculated in both the warfarin group and DOACs group with respect to gender differences. The adjusted HRs were converted to the natural logarithms and standard errors. The inverse variance method was used to incorporate random effect models.

## Results

### Study Selection

**FIGURE 1:**
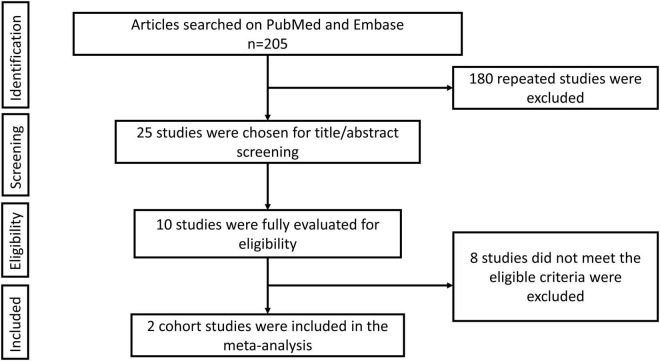
Retrieval flow chart of this meta-analysis.

The retrieval flow chart of this meta-analysis is shown in Figure 1. A total of **205** studies were acquired through online searching in the PubMed and Embase databases. After removing repeated investigations, 25 studies were chosen to develop title/abstract screening. Then 10 studies were evaluated in detail. On the basis of predefined criteria, finally, two eligible cohort studies were included in our meta-analysis (9, 15). Exhibited in Table 1 was the baseline information of patients in the included studies. A total of 24,434 individual participants (warfarin: *n* = 6,906; DOACs: *n* = 17,528) from two cohort studies were included in our meta-analysis. The data of included cohort studies were from Taiwan’s National Health Insurance Research Database (NHIRD) and Clinical Data Analysis and Reporting System (CDARS) managed by the Hong Kong Hospital Authority (HA). Their study periods were not less than 5 years and the medical use conditions that may influence patients’ DM risk were recorded in the baseline characteristic table. Both of these two studies could meet our screening criteria. For the quality assessment, the NOS scores of both included studies were ≥ 6 points. The number of included studies was less than 10. Thus, there was no need for publication bias assessment.

**TABLE 1:** Main baseline characteristics of DOACs and warfarin treated patients in the included cohort studies.

	Huang et al. (9)		Cheung et al. (15)			Warfarin
	DOACs	Warfarin	DOACs			
			Apixaban	Dabigatran	Rivaroxaban	
**Basal characteristics**						
Sample size, n	4,596	3,452	3,335	4,210	2,689	3,454
Age	70.9 (± 12.0)	70.6 (± 12.9)	78.1 (± 10.8)	74.4 (± 10.3)	74.9 (± 10.8)	72.9 (± 12.2)
Female Sex,%	40.0	39.2	50.9	47.9	47.7	44.9
Median follow-up duration	2.4 year	2.3 years	363 (106–648) days	363 (84–700) days	392 (98–730) days	222 (36–704) days
Data source	From Taiwan’s National Health Insurance Research Database (NHIRD)		Clinical Data Analysis and Reporting System (CDARS) managed by the Hong Kong Hospital Authority (HA)			
Country	Taiwan, China		Hong Kong, China			
Study period	5 years		6 years			
Outcomes	new-onset DM requiring treatment with an anti-diabetic drug		ICD-9-CM 250.xx including type 1 and type 2 diabetes or a prescription of anti-diabetic medication.			
**Comorbidities, n (%)**						
Congestive heart failure	766 (31.1)	737 (29.9)	636 (19.1)	576 (13.7)	413 (15.4)	741 (21.5)
Stroke	742 (30.1)	681 (27.6)	706 (21.2)	797 (18.9)	460 (17.1)	525 (15.2)
COPD	357 (14.5)	350 (14.2)	304 (9.1)	338 (8.0)	215 (8.0)	313 (9.1)
Fall	–	–	637 (19.1)	617 (14.7)	420 (15.6)	566 (16.4)
Fracture	–	–	299 (9.0)	282 (6.7)	214 (8.0)	251 (7.3)
Chronic liver disease/liver cirrhosis	55 (2.2)	56 (2.3)	16 (0.5)	14 (0.3)	4 (0.1)	23 (0.7)
Osteoporosis	–	–	65 (1.9)	51 (1.2)	41 (1.5)	37 (1.1)
Rheumatoid arthritis	20 (0.8)	22 (0.9)	39 (1.2)	36 (0.9)	22 (0.8)	29 (0.8)
Chronic kidney disease	292 (11.9)	280 (11.4)	79 (2.4)	42 (1.0)	49 (1.8)	187 (5.4)
Hypertension	1,616 (65.6)	1,619 (65.7)	–	–	–	–
Coronary artery disease	821 (33.3)	801 (32.5)	–	–	–	–
Hyperlipidemia	646 (26.2)	613 (24.9)	–	–	–	-
Dementia	142 (5.8)	136 (5.5)	–	–	–	–
Gout	265 (10.8)	251 (10.2)	–	–	–	–
Malignancy	185 (7.5)	194 (7.9)	–	–	–	–
**Medication use condition**						
ACE inhibitors	–	–	1,455 (43.6)	1,529 (36.3)	1,088 (40.5)	1,504 (43.5)
Beta blockers	1,472 (59.7)	1,502 (60.9)	1,959 (58.7)	2,490 (59.1)	1,649 (61.3)	2,019 (58.5)
Proton pump inhibitors	–	–	1,396 (41.9)	1,321 (31.4)	877 (32.6)	1,169 (33.8)
Systemic corticosteroids	–	–	324 (9.7)	341 (8.1)	222 (8.3)	377 (10.9)
Anti-depressants	–	–	202 (6.1)	199 (4.7)	128 (4.8)	162 (4.7)
Statins	143 (5.8)	134 (5.4)	–	–	–	–
Thiazides	199 (8.1)	191 (7.8)	–	–	–	–
Antipsychotics	143 (5.8)	141 (5.7)	–	–	–	–
Steroid	143 (5.8)	134 (5.4)	–	–	–	–
**Index year**						
2012	45 (1.8)	45 (1.8)	–	–	–	–
2013	476 (19.3)	476 (19.3)	–	–	–	–
2014	593 (24.1)	593 (24.1)	90 (2.7)	403 (9.6)	371 (13.8)	704 (20.4)
2015	694 (28.2)	694 (28.2)	256 (7.7)	578 (13.7)	525 (19.5)	727 (21.0)
2016	657 (26.7)	657 (26.7)	486 (14.6)	785 (18.6)	621 (23.1)	670 (19.4)
2017	–	–	886 (26.6)	1,045 (24.8)	547 (20.3)	630 (18.2)
2018	–	–	1,163 (34.9)	1,174 (27.9)	524 (19.5)	628 (18.2)
2019	–	–	454 (13.6)	225 (5.3)	101 (3.8)	95 (2.8)

*COPD, Chronic Obstructive Pulmonary Disease**;** DOACs, direct oral anticoagulants.*

### Crude Event Rates Between Direct Oral Anticoagulants vs Warfarin

The crude rates of the occurrence of the incident DM in AF patients treated by warfarin or DOACs were reported in both cohort studies, shown in Table 2. Compared with warfarin, the incidence of new-onset diabetes was relatively lower in DOACs treated group (6.78% vs 7.68%). Both coagulation factor Xa inhibitors apixaban (5.38% vs 6.14%), rivaroxaban (8.03% vs 8.05%) and thrombin inhibitor dabigatran (6.73% vs 8.49%) show the effectiveness in reducing the incidence of new-onset diabetes in AF patients, and this effect in coagulation factor Xa inhibitors combination group also exist (6.82% vs 7.23%).

**TABLE 2:** Pooled HRs of diabetes between DOACs vs. warfarin in patients with AF.

	DOACs	Dabigatran	Rivaroxaban	Apixaban	Rivaroxaban + Apixaban
Crude event rates	6.78% vs. 7.68%	6.73% vs. 8.49%	8.03% vs. 8.05%	5.38% vs. 6.14%	6.82% vs. 7.23%
HRs and 95% CIs	0.75 (0.68–0.82)	0.76 (0.66–0.88)	0.74 (0.64–0.86)	0.74 (0.60–0.92)	0.74 (0.66–0.84)
*P* value	<0.00001	0.001	0.0001	0.007	<0.00001
I^2^ statistic	0%	30%	1%	0%	0%

*AF, atrial fibrillation; HR, hazard ratio; CI, confidence interval; DOACs, Direct oral anticoagulants.*

### Adjusted Data of Outcomes Between Direct Oral Anticoagulants vs Warfarin

**FIGURE 2:**
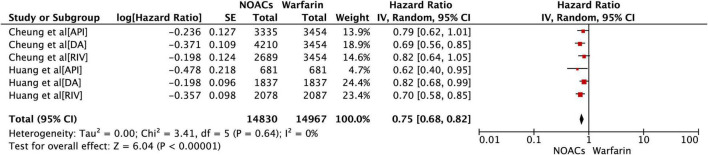
Comparing the primary outcomes of incident diabetes in DOACs vs. warfarin. DOACs, direct oral anticoagulants; HR, hazard ratio; CI, confidence interval; SE, standard error; IV, inverse of the variance.

Both included studies have reported the adjusted data of new-onset DM in AF patients treated by DOACs vs warfarin (9, 15). The outcomes displayed in Figure 2 were able to confirm that compared with warfarin, DOACs can reduce the risk of diabetes in AF patients (HR = 0.75, 95%CI: 0.68–0.82). Moreover, the outcomes of the gender subgroup according to the included studies are shown in Supplementary Figure 1. Compared with warfarin, the tendency of DOACs to reduce the incidence of new-onset diabetes can be observed in both male and female groups (male: HR = 0.73, 95%CI: 0.24–0.84; female: HR = 0.82, 95%CI: 0.68–0.99).

As shown in Table 2, the subgroup analysis of different types of DOAC were also analyzed in our study. All of the three evaluated DOACs associated with the decreased risk of inducing incident diabetes (Apixaban: HR = 0.74, 95% CI: 0.60–0.92; Dabigatran: HR = 0.76, 95% CI: 0.66–0.88; Rivaroxaban: HR = 0.74, 95% CI: 0.64–0.86). After pooling the data of rivaroxaban and apixaban, a similar effect could also be detected (HR = 0.74, 95% CI: 0.66–0.84), which confirms the reducing-effect of coagulation factor Xa inhibitors on the risk of new onset diabetes.

## Discussion

The primary findings of this study included (1) Compared with warfarin, DOACs including thrombin inhibitor dabigatran, coagulation factor Xa inhibitor rivaroxaban and apixaban could reduce the risk of new-onset diabetes in AF patients. (2) The DM-reduced effect of DOACs vs. warfarin can be observed in both male and female groups.

Compared with DOACs, warfarin has various limitations in the process of anticoagulant treatment. The changing international standardized ratio (INR) control and dose adjustment, various dietary or drug interactions (16, 17), narrow therapeutic window (17) result in the restrictions of warfarin in clinical use. Several meta-analyses and randomized controlled trials have reported the contrasts with the effectiveness and safety between DOACs and warfarin (18–20). The role of reducing AF and diabetes-associated risk factors including major bleeding, renal decline and cardiac valve calcification can be observed in the use of DOACs rather than warfarin (21–25).

Warfarin plays its role by antagonizing vitamin K, which is an important influence factor of glucose homeostasis and insulin sensitivity. In animal tissues, vitamin K homolog menaquinone-4 (MK-4) might act as an incretin-like nutrient and a cofactor of microsomal γ-glutamyl carboxylase (14, 26, 27). It contributes to the post-translational carboxylation process of transferring glutamate to γ-carboxyglutamate (Gla) residues of VKDP. Insulin production could be promoted by VKDP–osteoblast-specific secreted osteocalcin in a bone-pancreas endocrine loop to regulate glucose metabolism (14). The insulin resistance ameliorating effect of vitamin K was suggested through the inactivation of the NF-κβ signaling pathway to inhibit inflammatory responses and lipid-decreasing effect (11, 12). Considering the vitamin K antagonizing function of warfarin, it can influence the incidence of diabetes. However, the anticoagulation process of DOACs does not influence the vitamin K concentration in the circulatory system. Therefore, using DOACs compared with warfarin could reduce the risk of new-onset diabetes in AF patients.

In 2017, a novel drug betrixaban was approved by FDA as the fifth DOAC that can be used in clinic. With low renal clearance and minimal hepatic metabolism, betrixaban was considered particularly beneficial for patients with renal or hepatic dysfunction (28). However, the lack of an effective reversal agent makes betrixaban has a longer terminal half-life compared with other approved DOACs (28, 29). The impact of betrixaban on the risk of new-onset diabetes has not been evaluated in current studies, and the relevant results are expected to be supplemented in the future.

Our study was based on two cohort studies with a total sample size of 24,434, which is the most comprehensive and latest study according to the risk of DOACs vs. warfarin in inducing new-onset diabetes in AF patients. The result in the cohort study from Cheung et al. (15) proposed that only dabigatran was significantly associated with incident diabetes risk reduction, our results support that three of existing approved DOAC dabigatran, apixaban, and rivaroxaban with the function of reducing incident diabetic risk. At the same time, this effect in factor Xa inhibitors rivaroxaban and apixaban were not obvious, and our findings confirmed that all of these three drugs with the risk reduction ability. Also, in the outcomes of Cheung et al., only a specific gender of AF patients with the advantage of incident diabetic risk reduction in DOACs treatment, whereas the result of our investigation suggested that this effect could be observed in both male and female groups. In addition, the estimated crude events rates of new-onset diabetes were evaluated during our investigation process. Although available data is insufficient to support the effect of vitamin K in ameliorating prediabetes (the impaired glucose tolerance, fasting blood sugar, fasting serum insulin level would not be restored), the glucose and insulin levels of 2-h post-oral glucose tolerance test could be reduced by stable vitamin K support (10, 30, 31), which indicate that DOACs may not induce the rapid deterioration of prediabetes compared with warfarin. Also, the clinical trials have demonstrated that the new anti-diabetic drug sodium-glucose linked transporter inhibitors (SGLTi) with a beneficial effect on cardiovascular disease (32–34), regardless if diabetes exists or not (34). In the aspect of AF, SGLTi can counteract the production of cellular ROS in cardiomyocytes, which may change atrial remodeling and reduce the burden of AF (34). This suggests that the new anti-diabetic drug SGLTi and new oral anticoagulants DOAC may have similar effects on the prevention of new-onset diabetes during AF treatment.

The results of our study suggested that DOACs could reduce the risk of incident diabetes, which is probably more suitable for AF patients with a higher risk of new-onset diabetes. More prospective clinical data about the risk of incident DM in AF patients treated by DOACs and warfarin could prove our point.

## Limitations

This meta-analysis still had several limitations: (1) Only two cohort studies were included in our study, and the data were relatively limited, therefore the evaluation of the effect of edoxaban and betrixaban on incident diabetes was not supported. (2) The included population in our study only contain AF patients from Hong Kong and Taiwan, thus the evaluation of new-onset diabetes risk just considered Asian AF patients. (3) The subtypes of DM were not be distinguished in this study, whether DOACs have the same effect in reducing the risk of type 1 and type 2 diabetes in AF patients is still not clear. (4) The confounding factors cannot be completely excluded in observational studies. According to clinical guidelines, patients with rheumatic heart disease, congenital heart disease, or valve replacement surgery are more likely to be treated with warfarin rather than DOAC (9). This group that may induce selection bias was not completely excluded in our study. Future research could carry out propensity score matching on the basis of incorporating more data to minimize the impact of confounding factors.

## Conclusion

Our findings of current analysis suggested that treatment with DOACs compared with warfarin reduced the risk of new-onset diabetes in both male and female patients with AF.

## Data Availability Statement

The original contributions presented in the study are included in the article/Supplementary Material, further inquiries can be directed to the corresponding author/s.

## Author Contributions

All authors listed have made a substantial, direct, and intellectual contribution to the work, and approved it for publication.

## Conflict of Interest

The authors declare that the research was conducted in the absence of any commercial or financial relationships that could be construed as a potential conflict of interest.

## Publisher’s Note

All claims expressed in this article are solely those of the authors and do not necessarily represent those of their affiliated organizations, or those of the publisher, the editors and the reviewers. Any product that may be evaluated in this article, or claim that may be made by its manufacturer, is not guaranteed or endorsed by the publisher.

## Footnotes

1
https://community.cochrane.org/
