# Supplementary material for: Is the Risk of Diabetes Lower in Patients With Atrial Fibrillation Treated With Direct Oral Anticoagulant Compared to Warfarin?
Source: Front Cardiovasc Med. 2022 May 19;9:874795. doi: 10.3389/fcvm.2022.874795 (PMC9160370; doi:10.3389/fcvm.2022.874795)
Supplement: Supplementary file 1 [file Image_1.pdf]

Figure S1

Comparing the gender difference of incident diabetes in DOACs versus warfarin

Study or Subgroup	log[Hazard Ratio]	SE	Weight	Hazard Ratio IV, Random, 95% CI
**1.2.1 Males**				
Cheung et al[API+M]	−0.478	0.154	11.8%	0.62 [0.46, 0.84]
Cheung et al[DA+M]	−0.357	0.141	13.9%	0.70 [0.53, 0.92]
Cheung et al[RIV+M]	−0.357	0.164	10.5%	0.70 [0.51, 0.97]
Huang et al[NOAC+M]	−0.186	0.107	22.9%	0.83 [0.67, 1.02]
**Subtotal (95% CI)**			**59.1%**	**0.73 [0.64, 0.84]**
Heterogeneity: $Tau^2 = 0.00$; $Chi^2 = 2.73$, df = 3 (P = 0.44); $I^2 = 0\%$				
Test for overall effect: Z = 4.59 (P < 0.00001)				
**1.2.2 Females**				
Cheung et al[API+F]	0.02	0.198	7.3%	1.02 [0.69, 1.50]
Cheung et al[DA+F]	−0.371	0.166	10.3%	0.69 [0.50, 0.96]
Cheung et al[RIV+F]	−0.02	0.184	8.4%	0.98 [0.68, 1.41]
Huang et al[NOAC+F]	−0.301	0.136	14.9%	0.74 [0.57, 0.97]
**Subtotal (95% CI)**			**40.9%**	**0.82 [0.68, 0.99]**
Heterogeneity: $Tau^2 = 0.01$; $Chi^2 = 3.80$, df = 3 (P = 0.28); $I^2 = 21\%$				
Test for overall effect: Z = 2.09 (P = 0.04)				
**Total (95% CI)**			**100.0%**	**0.76 [0.69, 0.85]**
Heterogeneity: $Tau^2 = 0.00$; $Chi^2 = 7.52$, df = 7 (P = 0.38); $I^2 = 7\%$				
Test for overall effect: Z = 4.92 (P < 0.00001)				
Test for subgroup differences: $Chi^2 = 0.97$, df = 1 (P = 0.32), $I^2 = 0\%$				

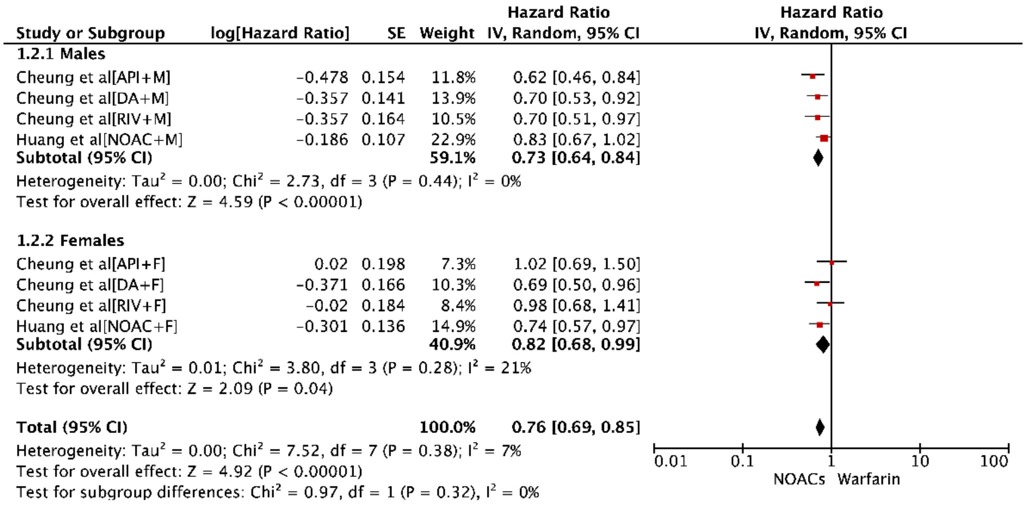

Abbreviations: DOACs=direct oral anticoagulants; HR=hazard ratio; CI=confidence interval; SE=standard error; IV=inverse of the variance.
